# Use of Preferred Source of Contraception Among Users of the Pill, Patch, and Ring in the US

**DOI:** 10.1001/jamanetworkopen.2024.39191

**Published:** 2024-10-21

**Authors:** Anu Manchikanti Gomez, Ariana H. Bennett, Alex Schulte, Jennet Arcara, Lisa Stern, Angela D. Aina, Jamie Bardwell, Denicia Cadena, Aisha Chaudhri, Laura Davis, Christine Dehlendorf, Brittni Frederiksen, Elizabeth Jones, Megan L. Kavanaugh, Catherine Labiran, Raegan McDonald-Mosley, Ellen Pliska, Whitney S. Rice, Ena Suseth Valladares, Cassondra Marshall

**Affiliations:** 1 Sexual Health and Reproductive Equity Program, School of Social Welfare, University of California, Berkeley; 2 Coalition to Expand Contraceptive Access, Washington, DC; 3 Black Mamas Matter Alliance, Atlanta, Georgia; 4 Converge, Ridgeland, Mississippi; 5 Independent Consultant, Albuquerque, New Mexico; 6 Illinois Caucus for Adolescent Health, Chicago; 7 Advocates for Youth, Washington, DC; 8 Person-Centered Reproductive Health Program, Departments of Family and Community Medicine, Obstetrics, Gynecology and Reproductive Sciences, and Epidemiology and Biostatistics; University of California, San Francisco; 9 KFF, San Francisco, California; 10 National Family Planning and Reproductive Health Association, Washington, DC; 11 Guttmacher Institute, New York, New York; 12 Independent consultant, Brooklyn, New York; 13 Power to Decide, Washington, DC; 14 Association of State and Territorial Health Officials, Arlington, Virginia; 15 Center for Reproductive Health Research in the Southeast, Rollins School of Public Health, Emory University, Atlanta, Georgia; 16 California Latinas for Reproductive Justice, Los Angeles; 17 School of Public Health, University of California, Berkeley

## Abstract

**Importance:**

Individuals who use contraceptive pills, patches, and rings must frequently interact with the health care system for continued and consistent use. As options for obtaining these methods expand, better understanding contraceptive users' preferences for source of contraception can help facilitate access.

**Objective:**

To describe use of preferred source of contraception and to understand associations between prior reproductive health care experiences and preference for traditional in-person sources vs alternative sources.

**Design, Setting, and Participants:**

This was a cross-sectional nationally representative survey study fielded in the US in 2022 through NORC's population-based AmeriSpeak panel. Eligible panelists were assigned female sex at birth, aged 15 to 44 years, not infecund, and not personally using permanent contraception. Of the screened panelists, 3150 were eligible; 3059 completed the survey (97%). The analysis sample included 595 individuals who currently used a pill, patch, or ring contraceptive. Data were analyzed from January 25, 2023, to August 15, 2024.

**Main Outcomes and Measures:**

Primary outcomes were use of any preferred source and use of most preferred source when most recently obtaining contraception. Sources included in-person care, telehealth, pharmacist-prescribed, online service, and over the counter. A binary variable captured whether respondents most preferred traditional, in-person contraceptive care vs an alternative approach. Prior reproductive health care experiences included missing a pill, patch, or ring because they couldn’t obtain it on-time and receipt of person-centered contraceptive counseling at most recent contraceptive visit. Sociodemographic characteristics and responses are presented as unweighted numbers with weighted prevalences.

**Results:**

In this study, 581 of 595 unweighted respondents (weighted prevalence, 95.9%) of the analytic sample identified as female, 256 unweighted respondents (weighted prevalence, 64.4%) were ages 15 to 29 years, and 448 unweighted respondents (weighted prevalence, 73.8%) had most recently obtained their contraception in-person. Only 197 unweighted respondents (weighted prevalence, 35.6%) selected in-person care as their most preferred source. Additionally, 296 unweighted respondents (weighted prevalence, 49.7%) had most recently obtained their method through any preferred source, and 227 unweighted respondents (weighted prevalence, 39.8%) had obtained it through their most preferred source. In adjusted regression analyses, respondents who previously reported being unable to get their method on time (adjusted odds ratio [aOR], 2.57; 95% CI, 1.36-4.87) had higher odds of preferring an alternative source vs traditional in-person care. Those who recently received person-centered contraceptive counseling (aOR, 0.59; 95% CI, 0.35-0.98) had lower odds of preferring an alternative source.

**Conclusions and Relevance:**

In this national study of individuals who used the pill, patch, and ring, most preferred alternative sources to obtain their contraception, and only half had most recently used a preferred source. These findings suggest that expansion of alternative contraceptive sources can better meet the needs of contraceptive users and support reproductive autonomy.

## Key Points

**Question:**

Are contraceptive pill, patch, and ring users in the US obtaining their method through a preferred source, and do they prefer traditional in-person care or alternative sources (eg, telehealth, over the counter)?

**Findings:**

In this survey study of 595 unweighted respondents, 49.7% of pill, patch, and ring users obtained their method from a preferred source, while 39.8% received it from their most preferred source. Over half (55.3%) did not select in-person care as a preferred source.

**Meaning:**

These findings suggest that many pill, patch, and ring users are not obtaining their methods from a preferred source, and most prefer alternative sources to obtain their contraception.

## Teaser

This survey study describes the use of preferred source of contraception and assesses associations between prior reproductive health care experiences and preference for traditional in-person sources or alternative sources.

## Introduction

Despite advances in method availability and insurance coverage, barriers to contraceptive access persist in the US, including logistical and interpersonal challenges,^1^ misinformation about contraception,^2,3,4^ and legal obstacles.^5,6,7^ Individuals who wish to continue use of short-acting, reversible contraception (SARC, including the pill, patch, and ring) must periodically engage with the health care system to renew prescriptions and obtain additional supplies. Given widespread use of SARC^8^ and requisite actions to continue use, access barriers have implications for reproductive autonomy.

How and where people receive contraception—including location, frequency, and clinician type—influence whether people receive care that meets their needs.^9,10,11^ In population-based data from Arizona, New Jersey, and Wisconsin, one-fourth of respondents expressed interest solely in traditional clinic-based contraceptive care, while most were interested in pharmacy-based and telehealth options.^12^ In another study, 40% of females who used contraception were not obtaining care through their preferred approach.^13^

Options for obtaining SARC have expanded beyond the traditional in-person, facility-based approach. Increasing evidence supports the safety and acceptability of alternative approaches, such as telehealth, pharmacist prescribing, and over-the-counter (OTC) provision.^14^ During the COVID-19 pandemic, telehealth (patient-clinician visits conducted virtually) for contraception became more common.^15,16^ Exclusively online, direct-to-consumer services for contraception have also proliferated.^17,18^ As of August 2023, 28 states and Washington DC authorized pharmacists to prescribe hormonal contraception.^19^ Additionally, an OTC progestin-only oral contraceptive pill became available in March 2024.^20,21^

Advancing health equity demands expanding access points for contraception. Alternative sources of care that allow individuals to obtain contraception without visiting a clinic can support improved access for people residing in remote areas, whose schedules limit attendance of in-person visits, and who prefer to avoid interactions with the health care system due to racism, other historic abuses, and prior low quality of care.^22,23,24,25^ Past and ongoing abuses include those specific to contraception (eg, forced and coerced sterilization, nonconsensual testing of contraceptives)^26,27,28^ and more general (eg, denials of care based on race, algorithmic biases, and poorer quality of interpersonal care).^29^ In a 2022 national survey, 67% of individuals assigned female sex at birth preferred to obtain contraception from a doctor’s office, but this proportion was substantially lower among respondents who were Black (19%) and Hispanic (18%), had low incomes (17%), and were uninsured (21%).^13^

Holistic, person-centered contraceptive access, including the source of care, is a crucial element of reproductive self-determination.^30,31,32^ Current approaches to measuring contraceptive access are not person-centered, focusing on use and services provided.^33,34,35,36^ In this study, we aimed to describe the use of preferred source of contraception among current SARC users as a person-centered metric of contraceptive access. Additionally, we examined associations between previous experiences obtaining contraception and preference for traditional vs alternative contraceptive sources, hypothesizing that those who had experienced access barriers or low-quality care would be more likely to prefer alternative sources.

## Methods

This survey study was reviewed and approved by the Committee for the Protection of Human Subjects at the University of California, Berkeley and the NORC Institutional Review Board. Survey methods were reported in accordance with American Association for Public Opinion Research (AAPOR) reporting guideline.

From January to March 2022, we fielded a cross-sectional nationally representative survey through NORC’s AmeriSpeak Panel as part of a project to develop person-centered contraceptive access metrics. We developed and modified survey questions about contraceptive access based on an extensive literature review and interviews conducted with 36 stakeholders from various sectors (eg, reproductive justice, clinical care, advocacy, research). To evaluate the comprehensibility and accessibility of key survey questions, we conducted 33 cognitive interviews with individuals meeting survey eligibility criteria. We convened the Person-Centered Contraceptive Access Metric Working Group reflecting similar diversity to stakeholder interviews to develop the metrics, which have been detailed elsewhere.^37^ Informed by the person-centered health care access^32^ and sexual and reproductive health (SRH) equity frameworks,^38^ the contraceptive access metrics address major gaps by centering self-identified need for contraceptive methods, services, and information.

The AmeriSpeak panel primary sampling frame is the 2010 NORC National Frame, a multistage probability sample constructed to represent the US household population. AmeriSpeak includes 54 001 members from more than 43 000 households, covering an estimated 97% of US households.^39^ NORC provided deidentified survey data. Eligibility criteria included being aged 15 to 44 years, assigned female sex at birth, not known to be sterile, and able to complete the survey in English or Spanish. NORC invited all panelists aged 18 to 44 years meeting sex criteria to read an online informed consent form and to consent by selecting yes. Those who consented completed the eligibility questionnaire and the full survey if eligible. For individuals aged 15 to 17 years, NORC first contacted parents; if they consented, NORC invited their child to assent and complete eligibility questions and the survey. Participants accessed the self-administered survey online with a median length of 25 minutes. Among eligible respondents who completed screening, the response rate was approximately 97%. NORC completed data quality checks, including removing respondents who completed the survey very quickly, refused to answer more than 50% of questions, and provided the same response across grid questions. Respondents received the equivalent of $8 in NORC points (NORC’s standard remuneration system for panelists) upon survey completion.

### Measures

Primary measures of interest are preferences for contraception sources and use of preferred sources. To assess preferences, we asked respondents, “If you could choose any way of getting a birth control prescription, how would you prefer to get it?” Response options were in-person at a doctor’s office or clinic; in-person at a hospital; from a telehealth (video or phone) appointment with a doctor’s office, clinic, or hospital; prescribed by a pharmacist; over the counter at a pharmacy or other store; from an online service that sends it directly; and in another way. Respondents could select multiple sources; those who did were prompted to select their most preferred source. For analysis, we combined the 2 in-person sources into a single category.

We also asked respondents, “The last time you got [method], how did you get it prescribed?” Responses were the same as above, excluding the OTC option (as there was not yet an OTC pill), and adding a response capturing asynchronous telehealth care: “contacted my clinician and they wrote a new prescription without seeing me for any kind of appointment first.”

To determine whether respondents had used a preferred source of contraception, we combined responses from the first question capturing their preferences and the second capturing where they had most recently obtained contraception to construct metrics that indicated receipt of contraception from any or the most preferred source. For example, if a respondent indicated multiple preferred sources (eg, in-person, pharmacist prescribing, and OTC) and had most recently obtained their method in-person, they were considered to have used any preferred source of contraception. However, if the same respondent most preferred receiving contraception OTC, they were not classified as using their most preferred source of contraception. If respondents indicated telehealth was a preferred source of contraception, they were classified as using a preferred source if they had mostly used synchronous (video or phone appointment) or asynchronous (contacting a clinician for a new prescription without an appointment) care (eAppendix in Supplement 1).

We asked respondents why they preferred their top source of contraception (16 response options consolidated into 10 categories). For bivariate and regression analyses, we created a binary variable that indicated whether respondents most preferred traditional in-person care vs an alternative source (ie, telehealth, pharmacist-prescribed, OTC, online service).

The survey also included sociodemographic items and SRH characteristics. NORC collected self-reported race and ethnicity as part of their standard demographic items for all AmeriSpeak panelists and provided it to the research team. Race and ethnicity categories included Asian or Pacific Islander only, Black only, Latina or Hispanic, White only, multiracial (not including Latina or Hispanic), or other race and ethnicity only. We do not have the specific race and ethnicity for the 7 respondents in this analytic sample who are categorized as other. We asked about experiences obtaining contraception, including missing a pill, patch, or ring because they were unable to get it on time; encountering challenges or delays in the past year; and whether they would have liked to change something about the process. Among respondents who had ever discussed contraception with a health care clinician, we included the person-centered contraceptive counseling measure.^40^ Respondents who received person-centered counseling rated their most recent contraceptive care clinician as excellent on: respecting them as a person, letting them say what mattered to them about their birth control, taking their preferences about your birth control seriously, and giving them enough information to make the best decision about their birth control method. We also asked about discriminatory experiences due to race, ancestry, or national origin.^41^ Those who had ever discussed contraception with a health care clinician were asked how often they had experienced the following types of discrimination in family planning settings during their lifetimes: being treated with less courtesy than other people; being treated with less respect than other people; receiving poorer service than other people; feeling like a doctor or nurse was not listening; a doctor or nurse assumed they were on welfare; a doctor or nurse made assumptions about the number of children they had; a doctor or nurse assumed they had multiple sexual partners; a doctor or nurse strongly encouraged them to use one method of birth control when they preferred another; and a doctor or nurse assumed they had a sexually transmitted disease, such as chlamydia, gonorrhea, genital warts, herpes, or HIV. We created a categorical variable describing the number of discrimination types ever experienced (ie, 0, 1 to 4, or 5 to 9).

### Sample

The analytic sample included individuals who currently used the contraceptive pill, patch, or ring and were not pregnant. We excluded 4 respondents reporting use of more than 1 of these methods in the past month, 11 respondents missing data on preferred source of contraception, and 7 respondents who most preferred another source that could not be categorized. The sociodemographic characteristics of the full sample and a bivariate analysis of those included vs excluded from the analytic sample are described in the eTable in Supplement 1.

### Statistical Analysis

We present descriptive statistics for sociodemographic and SRH characteristics, use and type of preferred contraceptive source, and reasons for preferences as unweighted numbers with weighted prevalences. We used Rao-Scott corrected χ^2^ tests to examine the association between whether respondents preferred a traditional in-person or alternative source, reasons for their preference, and previous experiences with contraceptive care. Additionally, we used multivariable logistic regression to examine associations between previous experiences and a preference for accessing care through an alternative contraceptive source vs traditional in-person care. Regressions adjusted for age, race and ethnicity, sexual orientation, education, insurance type, employment status, urbanicity, parental status, current pill use, reasons for SARC use, and duration of SARC use. Due to cell size issues, we were not able to conduct analyses considering the specific types of alternative sources. In all analyses, we used survey weights (constructed by NORC) to account for differences between the survey sample of 3059 individuals and the US population based on age, education, race and ethnicity, marital status, number of children in household, and age by race and ethnicity. We conducted all analyses using Stata version 17.0 (StataCorp). We applied svy commands to account for weighting and complex survey design. All reported proportions are weighted. Statistical significance was set at *P* < .05, and all tests were 2-sided. Data were analyzed from January 25, 2023, to August 15, 2024.

## Results

### Participant Characteristics

Among the full sample of 3059 individuals (unweighted), 2338 unweighted respondents (70.3%) had ever used the pill, patch, or ring (eTable in Supplement 1). Of the full sample, 595 unweighted respondents (21.7%) currently used these methods, comprising the analytic sample (data not shown). A lower proportion of parents and a higher proportion with commercial insurance were included in the analytic sample compared with those excluded (eTable in Supplement 1). Within the analytic sample, 126 unweighted individuals (weighted prevalence, 45.7%) were younger than 25 years (Table 1). Of the 595 individuals included in the sample, most identified as female (581 unweighted individuals; weighted prevalence, 95.9%), straight or heterosexual (495 unweighted individuals; weighted prevalence, 77.8%), and had commercial health insurance (455 unweighted individuals; weighted prevalence, 72.1%). Additionally, the study included 72 unweighted Black individuals (weighted prevalence, 13.1%), 105 unweighted Latina or Hispanic individuals (weighted prevalence, 20.7%), and 350 unweighted White individuals (weighted prevalence, 58.4%).

**Table 1.** Sociodemographic Characteristics of Pill, Patch, and Ring Users Aged 15 to 44 Years Among a 2022 National Sample (Unweighted N = 595)

Variable	Unweighted No. (weighted %)
Age, y	
15-17	46 (12.8)
18-24	80 (32.9)
25-29	130 (18.7)
30-34	136 (17.5)
35-39	121 (10.3)
40-44	82 (7.9)
Current gender identity	
Woman	581 (95.9)
Man	2 (0.3)
Transgender man	0
Genderqueer, nonbinary, fluid, nonconforming, agender, or another gender	2 (0.5)
More than 1 gender	8 (3.3)
Missing	2 (0.1)
Current sexual orientation	
Straight or heterosexual	495 (77.8)
Gay or lesbian	6 (2)
Bisexual	74 (17.6)
Queer	10 (1.5)
Something else	7 (0.8)
Missing	3 (0.4)
Race and ethnicity	
Asian or Pacific Islander only	35 (4.4)
Black only	72 (13.1)
Latina or Hispanic	105 (20.7)
Multiracial, not including Latina or Hispanic	26 (3)
Other race and ethnicity only^a^	7 (0.4)
White only	350 (58.4)
Relationship status	
Married, engaged, or in a serious relationship	355 (48.6)
In another type of relationship	133 (29.8)
Not in a relationship (including divorcing or separating)	105 (21.3)
Missing	2 (0.2)
Highest education completed	
Less than high school	61 (18.7)
High school or equivalent	69 (19.5)
Vocational or technical school, some college, or associates degree	184 (28.5)
Bachelor’s degree	183 (23.3)
Post graduate study or professional degree	98 (9.9)
Insurance type	
Commercial (eg, employer-based, direct purchase, health insurance exchange)	455 (72.1)
State Medicaid or CHIP	75 (14.3)
Other public insurance (including Medicare, military or VA, IHS)	20 (4)
None	23 (3.6)
Do not know	21 (5.8)
Missing	1 (0.2)
Working full-time	350 (51.5)
Urbanicity	
Urban	230 (37.8)
Suburban	276 (46.3)
Rural	89 (16)
Census region	
Northeast	93 (17.6)
Midwest	159 (24.8)
South	208 (35)
West	135 (22.6)
Born in the US	
No or prefer not to say	69 (9.6)
Yes	524 (90.3)
Missing	2 (0.2)

Abbreviations: CHIP, Children’s Health Insurance Program; IHS, Indian Health Service; VA, Veterans Affairs.

^a^ We do not have the specific race and ethnicity for the 7 respondents in this analytic sample who are categorized as other..

Most respondents were pill users (543 unweighted respondents; weighted prevalence, 92.3%), and 360 unweighted respondents (weighted prevalence, 62.1%) indicated they were currently using their preferred contraceptive method. Half (296 unweighted respondents; weighted prevalence, 49.7%) had most recently obtained their SARC method through any preferred source, and 227 unweighted respondents (weighted prevalence, 39.8%) had obtained it through their most preferred source (Table 2). Most commonly, respondents had last obtained their method in-person at a doctor’s office, clinic, or hospital (448 unweighted respondents; weighted prevalence, 73.8%), followed by an online service (48 unweighted respondents; weighted prevalence, 9.0%), asynchronous telehealth (44 unweighted respondents; weighted prevalence, 7.9%), synchronous telehealth (33 unweighted respondents; weighted prevalence, 5.2%), and prescribed by a pharmacist (13 unweighted respondents; weighted prevalence, 2.2%).

**Table 2.** Sexual and Reproductive Health Characteristics Among Pill, Patch, and Ring Users Aged 15 to 44 Years in a 2022 National Sample (Unweighted N = 595)

Variable	Unweighted No. (weighted %)
Use of preferred source of contraception	
Getting SARC through any preferred source	296 (49.7)
Getting SARC through most preferred source	227 (39.8)
Source used most recently to obtain SARC	
In-person at a doctor’s office, clinic, or hospital	448 (73.8)
Synchronous telehealth (video or phone) with a doctor’s office, clinic, or hospital	33 (5.2)
Contacted my clinician and they wrote a new prescription without seeing me for an appointment (asynchronous telehealth)	44 (7.9)
Prescribed by a pharmacist, without seeing a doctor or nurse first	13 (2.2)
From an online service that sends it directly	48 (9.0)
In another way	8 (1.5)
Missing	1 (0.3)
Current SARC method use	
Pill	546 (92.3)
Patch	12 (2.1)
Ring	37 (5.6)
Using preferred method of birth control^a^	
Yes	360 (62.1)
No	113 (20.3)
Uncertain	121 (17.2)
Missing	1 (0.4)
Currently using multiple methods of birth control	227 (42.8)
Reasons for pill, patch, or ring use^b^	
Pregnancy prevention	507 (84.2)
Regulate period	399 (71.6)
Alleviate period pain	254 (46.2)
Manage acne	119 (23.3)
Relieve mood swings	79 (15.0)
Relieve menstrual migraines	66 (11.4)
Manage PCOS	53 (10.9)
STI prevention	29 (7.4)
Manage endometriosis	21 (2.3)
Other	7 (1.3)
Manage fibroids	10 (1.1)
Using pill, patch, or ring for pregnancy prevention, other, or both	
Both pregnancy prevention and other reasons	385 (68.5)
Pregnancy prevention only	122 (15.7)
Other reasons only	88 (15.8)
Duration of pill, patch, or ring use	
Median (IQR), mo	41.2 (12.6-99.5)
0-6 mo	76 (12.8)
>6-12 mo	41 (7.8)
>12-24 mo	96 (19.1)
2-5 y	126 (22.5)
>5 y	221 (32.2)
Missing	35 (5.7)
Months supply of pill, patch, or ring most recently received, mo^c^	
1	120 (21.4)
2-3	378 (59.5)
4-6	46 (8.6)
7-12	46 (10.0)
Missing	4 (0.5)
Ever had penile-vaginal sex	531 (86.0)
Ever been pregnant	275 (31.5)
Parent	252 (26.5)
Ever missed a pill, patch, ring because you were unable to get it on time	193 (35.4)
Encountered challenges or delays obtaining the birth control method you wanted in the last 12 mo	128 (25.1)
Would have liked to change something about the process used to obtain birth control	90 (14.5)
Received person-centered contraceptive counseling from most recent health care clinician seen for birth control	
Yes	261 (38.1)
No	333 (61.7)
Missing	1 (0.3)
No. of types of discrimination ever experienced in a family planning setting	
0	296 (47.3)
1-4	174 (30.3)
5-9	115 (21.4)
Missing	10 (1.1)

Abbreviations: PCOS, polycystic ovary syndrome; SARC, short-acting reversible contraception; STI, sexually transmitted infection.

^a^ The term birth control was used in the survey measure itself and is included in the tables and manuscript text when referring specifically to the survey language. In other instances, the term contraception is used.

^b^ Responses not mutually exclusive.

^c^ Excludes segesterone acetate/ethinylestradiol 1-year ring user.

Respondents reported using their SARC method for pregnancy prevention (507 unweighted respondents; weighted prevalence, 84.2%), menstrual regulation (399 unweighted respondents; weighted prevalence, 71.6%), and to alleviate period pain (254 unweighted respondents; weighted prevalence, 46.2%); only 122 unweighted respondents (weighted prevalence, 15.7%) were exclusively using their SARC method for pregnancy prevention. Additionally, 448 unweighted respondents (weighted prevalence, 73.8%) had used their method for more than a year; 221 unweighted respondents (weighted prevalence, 32.2%) had used it for more than 5 years. A majority (498 unweighted respondents; weighted prevalence, 80.9%) had most recently received a 1-month to 3-month supply of their method. One hundred ninety-three unweighted respondents (weighted prevalence, 35.4%) had ever missed using their method because they were unable to get it on time; 128 unweighted respondents (weighted prevalence, 25.1%) had encountered challenges or delays obtaining contraception in the past year; and 90 unweighted respondents (weighted prevalence, 14.5%) wanted to change something about the process to obtain contraception. Just over a third (261 unweighted respondents; weighted prevalence, 38.1%) of respondents received person-centered contraceptive counseling from the most recent contraceptive care clinician. Half (289 unweighted respondents; weighted prevalence, 51.6%) had ever experienced discrimination in a family planning setting.

### Preferences for Sources of Contraception

Less than half (257 unweighted respondents; weighted prevalence, 44.7%) of respondents selected in-person, clinic-based care as a preferred source—followed by OTC (208 unweighted respondents; weighted prevalence, 32.4%), online services (165 unweighted respondents; weighted prevalence, 27.4%), pharmacist-prescribed (158 unweighted respondents; weighted prevalence, 26.4%), and telehealth (165 unweighted respondents; weighted prevalence, 25.4%) (Table 3). When considering their most preferred source of contraception, about one-third (197 unweighted respondents; weighted prevalence, 35.6%) of respondents selected traditional in-person, clinic-based care. Reasons for selecting their most preferred source included convenience (380 unweighted respondents; weighted prevalence, 63.1%), saving time (286 unweighted respondents; weighted prevalence, 48.0%), higher quality of care (trusting the clinician, feeling respected; 181 unweighted respondents; weighted prevalence, 33.0%), and affordability (lower cost, covered by insurance; 174 unweighted respondents; weighted prevalence, 32.5%) (Table 4). In bivariate analyses, those who selected convenience and saving time as reasons for their preference were more likely to most prefer an alternative source, whereas those who selected trusting and feeling respected by their clinician, usual location, and being able to ask questions were more likely to prefer traditional, in-person care.

**Table 3.** Preferred Sources of Contraception Among Pill, Patch, and Ring Users Aged 15 to 44 Years in a 2022 National Sample (Unweighted N = 595)^a^

Variable	Most preferred source, unweighted No. (weighted %)	A preferred source, unweighted No. (weighted %)
Traditional source		
In-person at a doctor’s office, clinic, or hospital	197 (35.6)	257 (44.7)
Alternative source		
Telehealth (video or phone) with a doctor’s office, clinic, or hospital	86 (13.2)	165 (25.4)
Prescribed by a pharmacist, without seeing a doctor or nurse first	69 (11.6)	158 (26.4)
Over the counter at a pharmacy or other store	134 (21.6)	208 (32.4)
From an online service that sends it directly	109 (18.0)	165 (27.4)

^a^ Responses not mutually exclusive.

**Table 4.** Reasons for Most Preferred Contraceptive Source and Relationship With Traditional vs Alternative Preference Among Pill, Patch, and Ring Users Aged 15 to 44 Years in a 2022 National Sample (Unweighted N = 595)

Reason(s) for most preferred source of contraception^a^	Unweighted No. (weighted %)	Preference for in-person of contraception, unweighted No. (weighted %)	Preference for alternative source, unweighted No. (weighted %)	*P* value
More convenient	380 (63.1)	38 (14.7)	342 (85.3)	<.001
Take less time	286 (48.0)	18 (7.3)	268 (92.7)	<.001
Trusting clinician or feeling respected	181 (33.0)	130 (72.1)	51 (27.9)	<.001
Cost or insurance	174 (32.5)	70 (40.7)	104 (59.3)	.23
Feel more in control	124 (24.3)	35 (31.5)	89 (68.6)	.44
Feel more private	116 (22.6)	40 (38.6)	76 (61.4)	.59
Usual location	121 (22.5)	69 (54.3)	52 (45.7)	<.001
Can ask questions	111 (21.1)	75 (64.3)	36 (35.7)	<.001
Method availability	61 (12.2)	23 (33.5)	38 (66.5)	.77
Another reason	63 (11.9)	35 (63.4)	28 (36.6)	.001

^a^ Respondents could select multiple reasons for their preference.

### Preferences for Traditional vs Alternative Sources of Contraception

In both bivariate and adjusted regression analyses, those who previously experienced issues related to obtaining contraception were more likely to prefer an alternative contraceptive source, while those who previously received higher quality care had reduced odds of preferring an alternative source (Table 5). Specifically, there were reduced odds of greatest preference for an alternative contraceptive source among those who had recently received person-centered contraceptive counseling (adjusted odds ratio [aOR], 0.59; 95% CI, 0.35-0.98) and those who had ever experienced 1 to 4 types of discrimination in a family planning setting (aOR, 0.45; 95% CI, 0.21-0.95) compared with 5 to 9 types.

**Table 5.** Associations Between Previous Care Experiences and Preference for Alternative Contraceptive Source Among Pill, Patch, and Ring Users Aged 15 to 44 Years in a 2022 National Sample (Unweighted N = 595)

Variable	Bivariate analysis			Adjusted logistic regression models	
	Preference for in-person source, unweighted No. (weighted %)	Preference for alternative source, unweighted No. (weighted %)	*P* value^a^	Preference for alternative source, aOR (95% CI)^b^	*P* value^a^
Ever missed a pill, patch, ring because unable to get on time	48 (26.5)	145 (73.5)	.03	2.57 (1.36-4.87)	.004
Encountered challenges or delays in getting the birth control method you wanted in the last 12 mo	26 (21.4)	102 (78.6)	.01	2.38 (1.15-4.96)	.02
Would have liked to change something about the process to obtain birth control	8 (12.9)	82 (87.1)	.005	4.32 (1.52-12.27)	.01
Received person-centered contraceptive counseling from most recent health care clinician seen for birth control	101 (37.3)	160 (62.7)	.63	0.59 (0.35-0.98)	.04
No. of types of discrimination ever experienced in a family planning setting					
0	102 (36.5)	194 (63.5)	.40	0.63 (0.32-1.27)	.20
1-4	62 (39.2)	112 (60.8)		0.45 (0.21-0.95)	.04
5-9	30 (28.2)	85 (71.8)		1 [Reference]	NA

Abbreviations: aOR, adjusted odds ratio; NA, not applicable; SARC, short-acting reversible contraception.

^a^ Regressions adjust for age, race and ethnicity, sexual orientation, education, insurance type, employment status, urbanicity, status as a parent, current pill user, reasons for SARC use, and duration of SARC use.

## Discussion

About half of respondents who were currently using the contraceptive pill, patch, and ring were not obtaining their methods through a preferred source of contraception. Notably, only 35.6% of respondents most preferred to receive care in-person, the dominant model of contraceptive care delivery in the US.^11,12,13^ Building upon previous research, we found that those who want a source that is more convenient and/or takes less time most prefer alternative sources. Those who previously experienced challenges accessing contraception were more likely to prefer alternative sources. Those who previously had received higher quality family planning care were less likely to prefer alternative sources, aligning with research documenting that nonperson-centered and discriminatory care alienates patients from the health care system.^37,42,43^

With 80% of SARC users in the sample most recently receiving only a 1- to 3-month supply, the norm of using these methods requires frequent engagements with the health care system. The short length of supplies dispensed presents many barriers for continued use and suggests failure to implement policies in 23 states that require Medicaid and/or commercial insurers to cover an extended supply of contraception (usually 12 months).^44^ It also indicates the presence of barriers constraining implementation of clinical guidelines that recommend routine dispensing of a 12-month supply of contraception.^44,45^ Clinician-level barriers include lack of awareness of the practice, belief that a 12-month supply would not be reimbursed by insurance, and implicit bias influencing decisions about which patients can and cannot handle a 12-month supply.^46^ A documented system-level factor is the need to update electronic health record templates so that a 12-month SARC supply is the default order length.^47^

To our knowledge, this is the first national study assessing the use of preferred source of contraception using a comprehensive range of sources, including OTC. We use population-based, nationally representative data, so findings have a high level of generalizability. We developed the metric use of preferred source of contraception using a process grounded in values of person-centered health care access^32^ and SRH equity.^38^

Policy strategies to expand contraceptive access must address implementation challenges that stand in the way of expanded access—for instance, those documented for implementing pharmacist prescribing and the requirement to have a prescription for OTC contraception to be covered by insurance.^48,49^ There is also a need for additional methods to be available OTC, assuming systems are in place for patients to obtain coverage without a prescription.^20,21^ In the meantime, concerted efforts are needed to educate the public on their contraceptive options so that they can successfully obtain and continue using different methods as desired. These data suggest that expansion of contraceptive sources may provide significant inroads for the ease, convenience, and successful use of popular contraceptive methods.

### Limitations

This study has limitations. We did not distinguish between preference for synchronous and asynchronous telehealth, which could result in improved measurement. While previous research indicates that preferred contraceptive source may differ across racial and ethnic groups, we lacked sufficient sample sizes to explore such differences. Our findings pertain only to individuals who currently used the pill, patch, and ring and do not address preferred source of contraception among the general population of individuals who could potentially use these methods, including those who may have faced the greatest access barriers and not been able to successfully obtain a desired SARC method, or individuals whose previous SARC use may have been supported by access to alternative sources. Finally, like all survey research, this study is limited in its inability to truly contextualize findings, even with the use of follow-up questions. Future studies investigating sources of contraception should examine how use and preferences change over time, especially as OTC access improves.

## Conclusions

Advancing SRH equity requires person-centered strategies for contraceptive access.^31^ The low level of preference for in-person care suggests that expanding contraceptive sources outside of traditional health care settings has a role in ameliorating barriers to access and can promote reproductive autonomy. Policymakers can also ensure equitable access to these methods by requiring all payers to reimburse clinicians in alternative settings, expand reimbursement for more types of clinicians, implement 1-year dispensing, and ensure that OTC methods are fully covered without a prescription.

## Notes

Supplement 1. **eAppendix.** Preferred Source of Contraception: Survey Items and Metric Construction **eTable.** Key Demographic Characteristics of Full Survey Sample and Bivariate Analysis of Respondents Included in the Analytic Sample vs Respondents Excluded From the Analytic Sample

Supplement 2.
Data Sharing Statement

## References

[1] Key K, Wollum A, Asetoyer C, . Challenges accessing contraceptive care and interest in over-the-counter oral contraceptive pill use among Black, Indigenous, and people of color: an online cross-sectional survey. Contraception. 2023;120:109950. doi:10.1016/j.contraception.2023.109950 36641098

[2] Varney S. Misinformation Clouds America’s Most Popular Emergency Contraception. KFF Health News. Accessed April 12, 2023. https://kffhealthnews.org/news/article/emergency-contraception-plan-b-private-equity-abortion-debate/

[3] Butler K. Inside anti-abortion groups’ campaign to sell women on unreliable birth control “alternatives.” Mother Jones. Accessed April 12, 2023. https://www.motherjones.com/politics/2022/09/inside-anti-abortion-groups-campaign-to-sell-women-on-unreliable-birth-control-alternatives/

[4] Malki LM, Patel D, Singh A. A mixed-methods analysis of women’s health misinformation on social media. In: Abdelnour Nocera J, Kristín Lárusdóttir M, Petrie H, Piccinno A, Winckler M, eds. Human-Computer Interaction – INTERACT 2023. Springer Nature Switzerland; 2023:419-428. doi:10.1007/978-3-031-42286-7_22

[5] Goldhill O. Supreme Court decision suggests the legal right to contraception is also under threat. STAT. Accessed April 12, 2023. https://www.statnews.com/2022/06/24/supreme-court-decision-suggests-the-legal-right-to-contraception-is-also-under-threat/

[6] Keith K. Supreme court upholds broad exemptions to contraceptive mandate—for now. Health Affairs. Accessed September 6, 2024. https://www.healthaffairs.org/content/forefront/supreme-court-upholds-broad-exemptions-contraceptive-mandate-now

[7] Kavanaugh ML, Friedrich-Karnik A. Has the fall of *Roe* changed contraceptive access and use: new research from four US states offers critical insights. Health Aff Sch. 2024;2(2):qxae016. "https://pubmed.ncbi.nlm.nih.gov/38756551/" doi:10.1093/haschl/qxae016 38756551 PMC10986283

[8] Frederiksen B, Ranji U, Long M. Women’s sexual and reproductive health services: key findings from the 2020 KFF Women’s Health Survey. KFF. Accessed June 25, 2021. https://www.kff.org/womens-health-policy/issue-brief/womens-sexual-and-reproductive-health-services-key-findings-from-the-2020-kff-womens-health-survey/

[9] Dixon S, Nancarrow SA, Enderby PM, Moran AM, Parker SG. Assessing patient preferences for the delivery of different community-based models of care using a discrete choice experiment. Health Expect. 2015;18(5):1204-1214. doi:10.1111/hex.12096 23809234 PMC5060844

[10] Feehan M, Walsh M, Godin J, Sundwall D, Munger MA. Patient preferences for healthcare delivery through community pharmacy settings in the USA: A discrete choice study. J Clin Pharm Ther. 2017;42(6):738-749. doi:10.1111/jcpt.12574 28627110

[11] Mirzaei M, Aspin C, Essue B, . A patient-centred approach to health service delivery: improving health outcomes for people with chronic illness. BMC Health Serv Res. 2013;13:251. doi:10.1186/1472-6963-13-251 23819721 PMC3706210

[12] Kavanaugh ML, Zolna MR. Where do reproductive-aged women want to get contraception? J Womens Health (Larchmt). 2023;32(6):657-669. doi:10.1089/jwh.2022.0406 37099807 PMC10277980

[13] Frederiksen B, Ranji U, Long M, Diep K. 2022. Contraception in the United States: a closer look at experiences, preferences, and coverage. KFF. Accessed November 11, 2022. https://www.kff.org/report-section/contraception-in-the-united-states-a-closer-look-at-experiences-preferences-and-coverage-findings/

[14] Williams RL, Meredith AH, Ott MA. Expanding adolescent access to hormonal contraception: an update on over-the-counter, pharmacist prescribing, and web-based telehealth approaches. Curr Opin Obstet Gynecol. 2018;30(6):458-464. doi:10.1097/GCO.0000000000000497 30299318

[15] Comfort AB, Rao L, Goodman S, . Assessing differences in contraceptive provision through telemedicine among reproductive health providers during the COVID-19 pandemic in the United States. Reprod Health. 2022;19(1):99. doi:10.1186/s12978-022-01388-9 35459218 PMC9026031

[16] Wilkinson TA, Kottke MJ, Berlan ED. Providing contraception for young people during a pandemic is essential health care. JAMA Pediatr. 2020;174(9):823-824. doi:10.1001/jamapediatrics.2020.1884 32379298 PMC7606730

[17] Frederiksen B, Gomez I, Salganicoff A. Contraception 2.0: Findings of a National Study of Online Contraception Platforms—Issue Brief. Kaiser Family Foundation. Accessed March 29, 2023. https://www.kff.org/report-section/contraception-2-0-findings-of-a-national-study-of-online-contraception-platforms-issue-brief/

[18] Frederiksen B. Who uses telecontraception and why: a closer look at clients of four telecontraception companies. KFF. Accessed April 14, 2023. https://www.kff.org/womens-health-policy/issue-brief/who-uses-telecontraception-and-why-a-closer-look-at-clients-of-four-telecontraception-companies/

[19] Guttmacher Institute. Pharmacist-prescribed contraceptives. Accessed January 26, 2024. https://www.guttmacher.org/state-policy/explore/pharmacist-prescribed-contraceptives

[20] Belluck P. First US over-the-counter birth control pill will be available soon. *New York Times*. Accessed March 10, 2024. https://www.nytimes.com/2024/03/04/health/opill-birth-control-nonprescription.html

[21] Office of the Commissioner. FDA approves first nonprescription daily oral contraceptive. FDA. Accessed July 14, 2023. https://www.fda.gov/news-events/press-announcements/fda-approves-first-nonprescription-daily-oral-contraceptive

[22] Rao L, Comfort AB, Dojiri SS, . Telehealth for contraceptive services during the COVID-19 pandemic: provider perspectives. Womens Health Issues. 2022;32(5):477-483. doi:10.1016/j.whi.2022.05.001 35691762 PMC9110325

[23] Sundstrom B, DeMaria AL, Ferrara M, Meier S, Billings D. “The closer, the better:” the role of telehealth in increasing contraceptive access among women in rural South Carolina. Matern Child Health J. 2019;23(9):1196-1205. doi:10.1007/s10995-019-02750-3 31228142

[24] Thompson TA, Ahrens KA, Coplon L. Virtually possible: using telehealth to bring reproductive health care to women with opioid use disorder in rural Maine. Mhealth. 2020;6:41. doi:10.21037/mhealth-19-237 33437837 PMC7793013

[25] Logan RG, Daley EM, Vamos CA, Louis-Jacques A, Marhefka SL. “When is health care actually going to be care?” the lived experience of family planning care among young black women. Qual Health Res. 2021;31(6):1169-1182. doi:10.1177/1049732321993094 33622078 PMC8114454

[26] Roberts DE. Killing the Black Body: Race, Reproduction, and the Meaning of Liberty. Vintage Books; 1999.

[27] Stern AM. Sterilized in the name of public health: race, immigration, and reproductive control in modern California. Am J Public Health. 2005;95(7):1128-1138. doi:10.2105/AJPH.2004.041608 15983269 PMC1449330

[28] Gutiérrez ER, Fuentes L. Population control by sterilization: the cases of Puerto Rican and Mexican-origin women in the United States. Lat Res Rev. 2009;7(3):85-100.

[29] Yearby R, Clark B, Figueroa JF. Structural racism in historical and modern US health care policy. Health Aff (Millwood). 2022;41(2):187-194. doi:10.1377/hlthaff.2021.01466 35130059

[30] Institute of Medicine. Crossing the quality chasm: a new health system for the 21st century. The National Academies Press. Accessed August 23, 2023. https://www.nap.edu/catalog/10027/crossing-the-quality-chasm-a-new-health-system-for-the 25057539

[31] Hart J, Crear-Perry J, Stern L. US sexual and reproductive health policy: which frameworks are needed now, and next steps forward. Am J Public Health. 2022;112(S5):S518-S522. doi:10.2105/AJPH.2022.306929 35767777 PMC10490305

[32] Levesque JF, Harris MF, Russell G. Patient-centred access to health care: conceptualising access at the interface of health systems and populations. Int J Equity Health. 2013;12:18. doi:10.1186/1475-9276-12-18 23496984 PMC3610159

[33] Bradley SEK, Casterline JB. Understanding unmet need: history, theory, and measurement. Stud Fam Plann. 2014;45(2):123-150. doi:10.1111/j.1728-4465.2014.00381.x 24931072 PMC4369378

[34] Senderowicz L. Contraceptive autonomy: conceptions and measurement of a novel family planning indicator. Stud Fam Plann. 2020;51(2):161-176. doi:10.1111/sifp.12114 32358789

[35] Holt K, Challa S, Alitubeera P, . Conceptualizing contraceptive agency: a critical step to enable human rights-based family planning programs and measurement. Glob Health Sci Pract. 2024;12(1):e2300299. doi:10.9745/GHSP-D-23-00299 38346841 PMC10906552

[36] Gomez AM, Reed RD, Bennett AH, Kavanaugh M. Integrating sexual and reproductive health equity into public health goals and metrics: a comparative analysis of healthy people 2030’s approach and a person-centered approach to contraceptive access. JMIR Public Health Surveill. 2024;10:e58009. doi:10.2196/58009 39163117 PMC11372330

[37] Gomez AM, Bennett AH, Arcara J, . Estimates of use of preferred contraceptive method in the United States: a population-based study. Lancet Reg Health Am. 2024;30:100662. doi:10.1016/j.lana.2023.100662 38304390 PMC10831268

[38] Stern L, Sterile H, Nguyen D, Moskosky S, Hart J. What is sexual and reproductive health equity and why does it matter for nurse practitioners? Womens Healthc (Doylestown). 2021;9(6):49-52. Accessed August 4, 2022. https://www.npwomenshealthcare.com/what-is-sexual-and-reproductive-health-equity-and-why-does-it-matter-for-nurse-practitioners/

[39] NORC. Technical overview of the AmeriSpeak panel, NORC’s probability-based household panel. NORC at the University of Chicago. Accessed September 6, 2024. https://amerispeak.norc.org/Documents/Research/AmeriSpeak%20Technical%20Overview%202019%2002%2018.pdf

[40] Person-centered contraceptive counseling measure. Partnership for Quality Measurement. Accessed May 31, 2023. https://p4qm.org/measures/3543

[41] Thorburn Bird S, Bogart LM. Birth control conspiracy beliefs, perceived discrimination, and contraception among African Americans: an exploratory study. J Health Psychol. 2003;8(2):263-276. doi:10.1177/1359105303008002669 22114130

[42] Kavanaugh ML, Pliskin E, Hussain R. Associations between unfulfilled contraceptive preferences due to cost and low-income patients’ access to and experiences of contraceptive care in the United States, 2015-2019. Contracept X. 2022;4:100076. doi:10.1016/j.conx.2022.100076 35620731 PMC9126850

[43] Gómez AM, Wapman M. Under (implicit) pressure: young Black and Latina women’s perceptions of contraceptive care. Contraception. 2017;96(4):221-226. doi:10.1016/j.contraception.2017.07.007 28756187

[44] Extended supply of contraception. Power to Decide. Accessed March 29, 2023. https://powertodecide.org/what-we-do/information/resource-library/extended-supply-contraception

[45] Gavin L, Moskosky S, Carter M, ; Centers for Disease Control and Prevention (CDC). Providing quality family planning services: recommendations of CDC and the US Office of Population Affairs. MMWR Recomm Rep. 2014;63(RR-04):1-54. 24759690

[46] Qasba NT, Dowd P, Bianchet E, Goff SL. A qualitative study of clinicians’ perspectives on a law that allows for a 12-month supply of short-acting contraceptives in Massachusetts: barriers and facilitators to implementation. Health Serv Res. 2023;58(2):498-507. doi:10.1111/1475-6773.14105 36414429 PMC10012237

[47] Uhm S, Chen MJ, Cutler ED, Creinin MD. Twelve-month prescribing of contraceptive pill, patch, and ring before and after a standardized electronic medical record order change. Contraception. 2021;103(1):60-63. doi:10.1016/j.contraception.2020.10.011 33098853 PMC7736567

[48] Gómez AM. Availability of pharmacist-prescribed contraception in California, 2017. JAMA. 2017;318(22):2253-2254. doi:10.1001/jama.2017.15674 29234797 PMC5820738

[49] Gomez AM, McCullough C, Fadda R, . Facilitators and barriers to implementing pharmacist-prescribed hormonal contraception in California independent pharmacies. Women Health. 2020;60(3):249-259. doi:10.1080/03630242.2019.1635561 31264530

