# Supplementary material for: Use of Preferred Source of Contraception Among Users of the Pill, Patch, and Ring in the US
Source: JAMA Netw Open. 2024 Oct 21;7(10):e2439191. doi: 10.1001/jamanetworkopen.2024.39191 (PMC11581482; doi:10.1001/jamanetworkopen.2024.39191)
Supplement: Supplement 1. — eAppendix. Preferred Source of Contraception: Survey Items and Metric Construction eTable. Key Demographic Characteristics of Full Survey Sample and Bivariate Analysis of Respondents Included in the Analytic Sample vs Respondents Excluded From the Analytic Sample [file jamanetwopen-e2439191-s001.pdf]

Supplemental Online Content

Gomez AM, Bennett AH, Schulte A, et al. Use of preferred source of contraception among users of the pill, patch, and ring in the US. *JAMA Netw Open*. 2024;7(10):e2439191. doi:10.1001/jamanetworkopen.2024.39191

eAppendix. Preferred Source of Contraception: Survey Items and Metric Construction
eTable. Key Demographic Characteristics of Full Survey Sample and Bivariate Analysis of Respondents Included in the Analytic Sample vs Respondents Excluded From the Analytic Sample

This supplemental material has been provided by the authors to give readers additional information about their work.

© 2024 Gomez AM et al. *JAMA Network Open.*

eAppendix 1. Preferred Source of Contraception: Survey Items and Metric Construction

Survey Items

Preferred sources of contraception

1. If you could choose any way of getting a birth control prescription, how would you prefer to get it? S*elect all that apply.*

RESPONSE OPTIONS:

1. In-person at a doctor's office or clinic [1]
2. In-person at a hospital[1]
3. From a telehealth (video or phone) appointment with a doctor's office, clinic, or hospital
4. Prescribed by a pharmacist, without seeing a doctor or nurse first
5. Over the counter at a pharmacy or other store (no prescription from any source needed)
6. From an online service that sends it directly (like Nurx) [2]
7. In another way

1a. [*If response* #7]: In response to the previous question, you said "In another way." Please specify how you would prefer to get a birth control prescription: [*Write-in response*]

1b. [*If >1 response to Q2 above*]: Which way of getting birth control would you most prefer?

Most recent source of contraception

2. The last time you got [*insert current method*], how did you get it prescribed?

RESPONSE OPTIONS:

1. In-person at a doctor's office or clinic[1]
2. In-person at a hospital[1]
3. From a telehealth (video or phone) appointment with my regular doctor's office, clinic, or hospital [3]
4. Contacted my provider and they wrote a new prescription without seeing me for any kind of appointment first
5. Prescribed by a pharmacist, without seeing a doctor or nurse first
6. From an online service that sends it directly to me (like Nurx)
7. In another way

2a. [*If response #7*]: In response to the previous question, you said "In another way." Please specify how you got [*insert current method*] prescribed: [*Write-in response*]

[1] For the metric, response options 1 and 2 were combined as "In-person at a doctor's office, clinic, or hospital."

[2] The original version of this question included "or the Pill Club" as an example along with Nurx in response option 6 in both questions above. However, as the Pill Club is no longer in business, we suggest removing it from the response option.

[3] For the metric, response options 3 (synchronous telehealth) and 4 (asynchronous telehealth) were combined under a single telehealth category for source of most recent contraception.

© 2024 Gomez AM et al. *JAMA Network Open.*

Metric Construction

<u>Receipt of contraception from *any* preferred source</u> = most recently obtaining the pill, patch, or ring from *any* of their preferred sources.

This was determined based on a match between responses for questions 1 and 2, as described below. For example, if a respondent (1) prefers in-person care, telehealth, and over-the counter access and (2) most recently obtained their contraception via telehealth, they were considered to have used *any* of their preferred sources of contraception.

<u>Receipt of contraception from *most* preferred source</u> = most recently obtaining the pill, patch, or ring from their *most* preferred source.

This was determined based on a match between questions 1/1b and 2. If only one preferred source was selected, the match was based on questions 1 and 2. If more than one preferred source was selected, the match was based on questions 1b and 2. For example, if a respondent (1) prefers in-person care, telehealth, and over-the counter access; (1b) has the greatest preference for over-the-counter access; and (2) most recently obtained their contraception via telehealth, they were not considered to have used their *most* preferred source of contraception.

Match between preferred source of contraception and most recent source of contraception response options

1/1b. Preferred sources of contraception	*2. Most recent source of contraception*
In-person at a doctor's office, clinic, or hospital	In-person at a doctor's office, clinic, or hospital
From a telehealth (video or phone) appointment with a doctor's office, clinic, or hospital	From a telehealth (video or phone) appointment with my regular doctor's office, clinic, or hospital<br><br>OR<br><br>Contacted my provider and they wrote a new prescription without seeing me for any kind of appointment first
Prescribed by a pharmacist, without seeing a doctor or nurse first	Prescribed by a pharmacist, without seeing a doctor or nurse first
Over-the-counter at a pharmacy or other store (no prescription from any source needed)	*No match, as over-the-counter oral contraception was not available at the time of the survey*
From an online service that sends it directly	From an online service that sends it directly

eTable 1. Key demographic characteristics of full survey sample (unweighted N=3,059) and bivariate analysis of respondents included in the analytic sample (unweighted n=595) vs. respondents excluded from the analytic sample (unweighted n= 2464)

	Full sample, Unweighted n (Weighted %)	Included in analytic sample, Unweighted n (Weighted %)	Excluded from analytic sample, Unweighted n (Weighted %)	*p value*[a]
Ever used pill, patch, or ring				----[b]
Yes	2338 (70.3)	595 (100)	1743 (62.1)	
No	718 (29.5)	0 (0.0)	718 (37.7)	
Missing	3 (0.2)	0 (0.0)	3 (0.23)	
Age				0.06
15-17	202 (11.8)	46 (12.8)	156 (11.5)	
18-24	321 (27.1)	80 (32.9)	241 (25.5)	
25-29	654 (21.2)	130 (18.7)	524 (21.9)	
30-34	749 (18.3)	136 (17.5)	613 (18.6)	
35-39	655 (12.2)	121 (10.3)	534 (12.8)	
40-44	478 (9.4)	82 (7.9)	396 (9.8)	
Highest education completed				0.69
Less than high school	289 (18.1)	61 (18.7)	228 (18.0)	
High school or equivalent	376 (20.4)	69 (19.5)	307 (20.7)	
Vocational or technical school, some college, or associates degree	1051 (30.8)	184 (28.5)	867 (31.4)	
Bachelor's degree	871 (20.9)	183 (23.3)	688 (20.2)	
Post graduate study or professional degree	472 (9.8)	98 (9.9)	374 (9.8)	
Race and ethnicity				0.07
Asian or Pacific Islander only	203 (7.1)	35 (4.4)	168 (7.8)	
Black only	435 (14.9)	72 (13.1)	363 (15.4)	
Latina or Hispanic	541 (21.1)	105 (20.7)	436 (21.2)	
Multiracial, not including Latina or Hispanic	153 (3.3)	26 (3.0)	127 (3.4)	
Other race and ethnicity only	41 (0.7)	7 (0.4)	34 (0.8)	
White only	1686 (53.0)	350 (58.4)	1336 (51.5)	
Parent				<0.001***
Yes	1641 (38.4)	252 (26.5)	1389 (41.7)	
No	1404 (61.1)	341 (73.4)	1063 (57.7)	
Missing	14 (0.5)	2 (0.1)	12 (0.6)	
Insurance type				0.001**
Commercial (e.g., employer-based, direct purchase, health insurance exchange)	2073 (62.5)	455 (72.1)	1618 (59.8)	
State Medicaid or CHIP	521 (19.9)	75 (14.3)	446 (21.4)	
Other public insurance (including Medicare, military/VA, IHS)	132 (4.5)	20 (4.0)	112 (4.6)	
None	196 (6.7)	23 (3.6)	173 (7.5)	
Don't know	110 (5.3)	21 (5.8)	89 (5.1)	
Missing	27 (1.2)	1 (0.2)	26 (1.4)	

© 2024 Gomez AM et al. *JAMA Network Open.*

a. p value reports statistical significance of chi-squared test for the distribution of each characteristic between those included vs. excluded from the analytic sample. *p < 0.05, **p < 0.01, ***p < 0.001

b. Chi-square test was not used due the structural zero cell size, as there were no respondents reflected in the “no” or “missing” cells due to the inclusion requirements for the analytic sample.

© 2024 Gomez AM et al. *JAMA Network Open.*
