# Supplementary material for: Use of Preferred Source of Contraception Among Users of the Pill, Patch, and Ring in the US
Source: JAMA Netw Open. 2024 Oct 21;7(10):e2439191. doi: 10.1001/jamanetworkopen.2024.39191 (PMC11581482; doi:10.1001/jamanetworkopen.2024.39191)
Supplement: Supplement 2. — Data Sharing Statement [file jamanetwopen-e2439191-s002.pdf]

Data Sharing Statement

Gomez. Use of Preferred Source of Contraception Among Users of the Pill, Patch, and Ring in the US. *JAMA Netw Open*. Published October 21, 2024.
doi:10.1001/jamanetworkopen.2024.39191

Data
Data available: Yes
Data types: Deidentified participant data
How to access data: The data and codebook will be publicly available from the Open Science Framework at https://osf.io/wvb5m/.
When available: beginning date: 01-01-2025

Supporting Documents
Document types: Statistical/analytic code
How to access documents: https://osf.io/wvb5m/
When available: beginning date: 01-01-2025

Additional Information
Who can access the data: De-identified data will be publicly available.
Types of analyses: For any purpose.
Mechanisms of data availability: Data will be publicly available at the OSF repository.
